# Breakpoints and deleted genes identification of ring chromosome 18 in a Chinese girl by whole-genome low-coverage sequencing: a case report study

**DOI:** 10.1186/s12881-016-0307-1

**Published:** 2016-07-22

**Authors:** Hui Yao, Chuanchun Yang, Xiaoli Huang, Luhong Yang, Wei Zhao, Dan Yin, Yuan Qin, Feng Mu, Lin Liu, Ping Tian, Zhisheng Liu, Yun Yang

**Affiliations:** Wuhan Medical Care Center for Women and Children, Wuhan, 430015 China; BGI-Wuhan, Wuhan, 430075 China; BGI-Shenzhen, Shenzhen, 518083 China; Department of Obstetrics and Gynecology, The Second Affiliated Hospital of Zhengzhou University, Zhengzhou, 450052 China

**Keywords:** Ring chromosome, Whole-genome low-coverage sequencing, Detailed breakpoints, Detailed diagnosis

## Abstract

**Background:**

Ring chromosome 18 [r(18)] is formed by 18p- and 18q- partial deletion and generates a ring chromosome. Loss of critical genes on each arm of chromosome 18 may contribute to the specific phenotype, and the clinical spectrum varieties may heavily depend on the extent of the genomic deletion. The aim of this study is to identify the detailed breakpoints location and the deleted genes result from the r18.

**Case presentation:**

Here we describe a detailed diagnosis of a seven-year-old Chinese girl with a ring chromosome 18 mutation by a high-throughput whole-genome low-coverage sequencing approach without karyotyping and other cytogenetic analysis. This method revealed two fragment heterozygous deletions of 18p and 18q, and further localized the detailed breakpoint sites and fusion, as well as the deleted genes.

**Conclusions:**

To our knowledge, this is the first report of a ring chromosome 18 patient in China analyzed by whole-genome low-coverage sequencing approach. Detailed breakpoints location and deleted genes identification help to estimate the risk of the disease in the future. The data and analysis here demonstrated the feasibility of next-generation sequencing technologies for chromosome structure variation including ring chromosome in an efficient and cost effective way.

**Electronic supplementary material:**

The online version of this article (doi:10.1186/s12881-016-0307-1) contains supplementary material, which is available to authorized users.

## Background

Ring chromosome 18 [r(18)] is formed from breakage of both ends of the chromosome and the break ends generate a ring chromosome [1]. Individuals with r(18) have 18p and 18q partial deletions and according phenotype, such as microcephaly, mental deficiency, hypotonia, and congenital heart defects [2, 3]. Short stature, microcephaly, mental deficiency, craniofacial dysmorphism and extremity abnormalities are the most commonly reported features in patients with r(18). The phenotype with r(18) syndrome is highly variable and depends on the combination of 18p- syndrome and 18q- syndrome. Loss of critical genes on each arm of chromosome 18 may contribute to the specific symptoms, and the clinical spectrum varieties may heavily depend on the extent of the genomic deletion [4].

Whole-genome low-coverage sequencing has been reported previously by our group to accurately detect chromosomal structural variation-associated breakpoints and affected region without cytogenetic analysis on patients [5].

In the current study, we applied whole-genome low-coverage sequencing to characterize the ring chromosome 18 mutation at a molecular level in a Chinese young girl for the first time. We described the full profile of clinical examination, genetic characterizations, and clinical treatment report. We localized the genomic breakpoints as well as identified the deleted genes. The deletion of the genes and detailed breakpoint identified help to understand the genotype- correlation and estimate the risk of the disease in the future.

## Case presentation

The patient was born to non-consanguineous at the year of 2006. The patient was born at 40 weeks gestation with a birth weight of 3,050 g and length of 49 cm. At 2 years of age, she was found shorter than children of the same age. In April 2008, she was diagnosed hypothyroidism in the local clinic. Replacement of thyroid hormone (levothyroxine) was started for the treatment of autoimmune hypothyroidism. Unregular treatment lasted one year and discontinue by parents themselves.

At 6 and half years of age (March,2013), she came to our hospital for short stature. At the time of our first evaluation, she had a short stature problem (height: 90.7 cm [−6.0SD, equivalently 50 percentile of 2–2.5 years old], weight: 12.0 kg [<3 percentile, equivalently 50 percentile of 2–2.5 years old]). The general examination phenotypes of this patient include intellectual disability with IQ = 70, hypoactive, poor appetite, hypotonia, short neck without webbing, short fingers and toes, much shorter fifth finger, sparse hair and dry skin. She had dry stool once every 1 ~ 3 days. No goiter, lymphadenopathy or hepatosplenomegaly were noted.

**Fig. 1:**
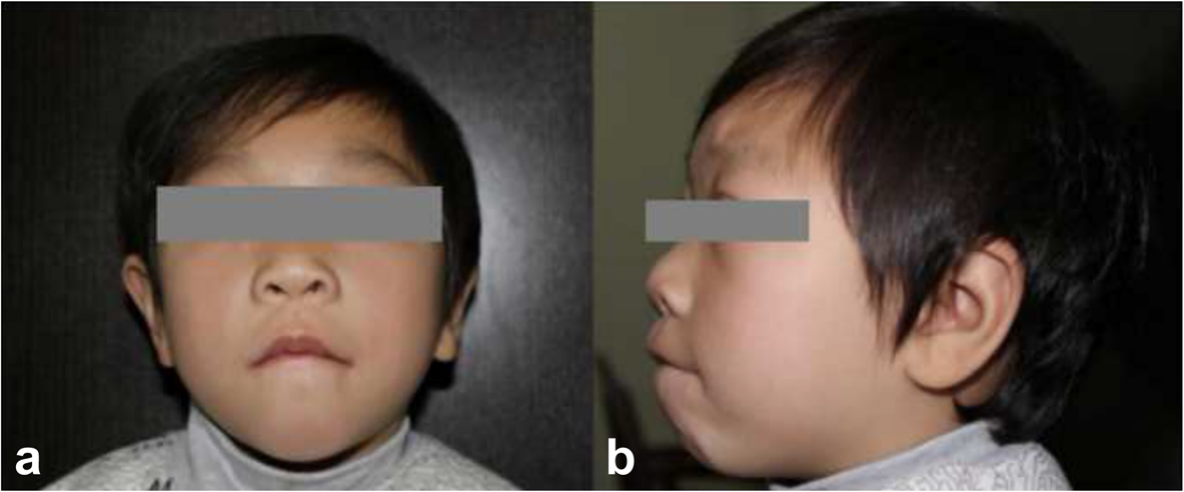
Abnormalities of the craniofacial appearance. Facial appearance of the patient at age 7, showing flat midface, puffy eyelids, hypertelorism, epicanthic fold, flat nasal bridge, and micrognathia. **a** frontal view. **b** lateral view

The facial appearance of the patient was including flat midface, puffy eyelids, hypertelorism, epicanthic fold, flat nasal bridge, and micrognathia. Wide mouth, downturned corners of mouth, thick lips, large protruding ears ptosis and upslanting palpebral ptosis were also noted (Fig. 1). High narrow palate and several cavities in teeth were observed. In addition, she suffered from bronchitis and otitis media frequently, without serious infections. Auscultation revealed no heart murmur and normal respiratory sounds.

Serological examination results showed normal liver and kidney functions but abnormal thyroid function, which prompted central autoimmune hypothyroidism and autoimmune thyroiditis. The thyroid auto antibodies were positive. Both TPO-Ab and TG-Ab were extremely high. The levels of IGF-1 and IGF-BP3 decreased drastically. IgA was slightly increased. E2、PROG、PRL and TESTO were all normal (data not show). Flow cytometry detection of T cell subgroup revealed that CD3 and CD8 + T were slightly higher (Additional file 1: Table S1). After euthyrox therapy, her total cholesterol and the triglyceride were back to normal levels, but the lipoprotein-α was still high (494.2 mg/l, reference range: 0-300 mg/l), the IGF-1 still low (29.8 ng/ml, reference range:64-345 ng/ml).

The abdominal color ultrasound results showed normal liver, uterus and ovaries. The sizes of both kidneys were smaller than normal. (left kidney: 6.2 cm × 2.8 cm, right kidney: 5.7 cm × 2.4 cm). The thyroid color ultrasound revealed that the thyroid was enlarged and its echo was not uniform. The thyroid isthmus was 0.5 cm thick (left lobe thyroid: 2.9 cm × 1.0 cm × 1.2 cm, right lobe thyroid: 3.3 cm × 1.0 cm × 1.3 cm) accompanied with uneven internal spots and echoes, like a network. The cardiac color ultrasound showed that the structure, shape and valves of the heart had no obvious abnormality.

**Fig. 2:**
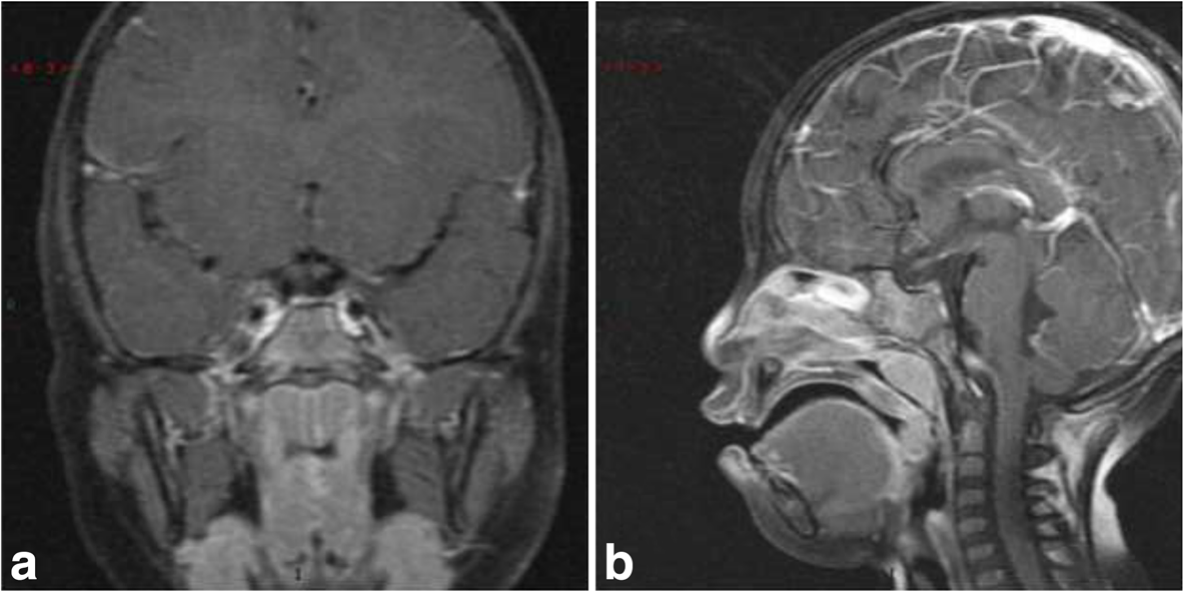
Pituitary gland on MRI. The MRI image results revealed that the Pituitary height was 1.0 mm, much smaller than the normal size, and the neurohypophysis was not seen clearly, which indicated pituitary dysplasia. **a** Coronal MRI scan of Pituitary Gland. **b** Sagittal MRI scan of Pituitary Gland

The MRI image results revealed that the Pituitary height was 1.0 mm, much smaller than the normal size, and the neurohypophysis was not seen clearly, which indicated pituitary dysplasia (Fig. 2).

**Fig. 3:**
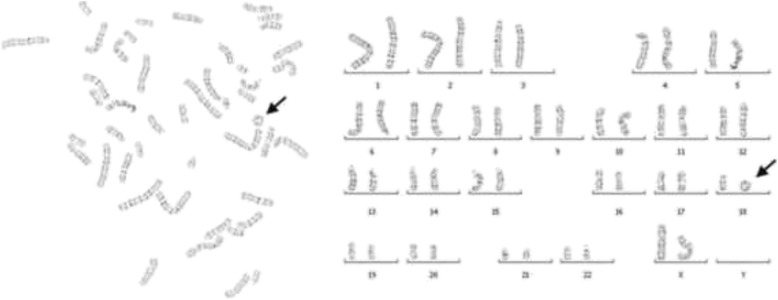
Patient’s Karyotype analysis result by G-banding technique. The result of conventional karyotyping showed 46, XX, r(18) (*arrow indicated*)

Following informed consent, hromosomal analysis was performed on peripheral blood lymphocyte cultures. The result of conventional karyotyping was 46, XX, r (18) (Fig. 3). No chromosomal anomaly was detected in either of parent by karyotyping analysis (data not shown).

Patient’s genomic DNA was extracted from peripheral blood using Qiagen DNA extraction kit and then was used to construct DNA libraries and to do sequencing assay including base calling. After removing reads containing sequencing adaptors and low quality reads, the high quality pair-end reads were aligned to the NCBI human reference genome (hg19, GRCh37.1) using SOAP2 [6]. Only uniquely mapped reads were remained in the following analysis.

The ring chromosome variation could be discovered using chimeric read pairs, which are paired-end reads that mapped to two different chromosomes.. The detail steps are listed in our previous published study [5].

**Fig. 4:**
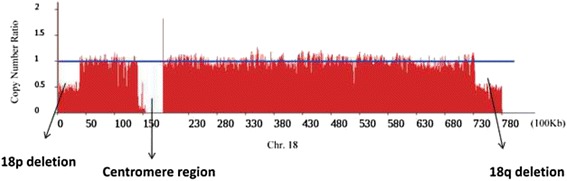
The Copy Number Ratio of Chromosome 18. The blue line indicates the normal diploid copy number ratio, and the window size is 5 kb. Both ends of chromosome 18 has a partial deletion, the copy number ratio is 0.5 showed heterozygous terminal deficiency. Centromere starts from 150Kb to 180Kb

**Fig. 5:**
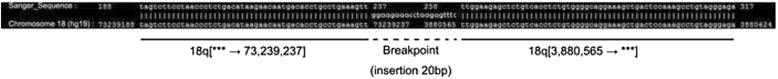
The Sanger sequence alignment around the breakpoints of chromosome 18. We found there are a 20 bp insertion between the fusion breakpoints

Finally, we identified two partial deletions which are a portion of 18p from 1 bp to nearly 3,881,000 bp (3.88 Mb), and a portion of 18q from nearly 73,239,191 bp to terminal (4.83 Mb) base on the bioinformatics results. Both ends of chromosome 18 showed heterozygous terminal deficiency (Fig. 4). The remaining sequence of chromosome 18 generated a ring from breakage and subsequent fusion of both chromosome arms. The two breakpoints located in 18p11.31 band and 18q23 band respectively. The detailed breakpoint sites were validated to be at 3,880,565 bp and at 73,239,237 bp of chr18 respectively by Sanger sequencing. Besides, we also found a 20 bp insertion between the fusion breakpoints (Fig. 5). There were 19 genes deleted at chromosome 18 (pter → p11.31) and 12 genes deleted at chromosome 18 (q23 → qter) (Tables 1 and 2).

**Table 1:** Genes and their genomic location within the deleted segment at 18p

Gene Symbol	Gene ID	Chromosome	Start Position	End Position	Strand
USP14	NM_001037334	chr18	158482	213739	+
	NM_005151				
THOC1	NM_005131	chr18	214519	268059	-
COLEC12	NM_130386	chr18	319354	500729	-
CETN1	NM_004066	chr18	580368	581524	+
	NM_014410				
	NM_199167				
C18orf56	NM_001012716	chr18	649619	658340	-
TYMS	NM_001071	chr18	657603	673499	+
ENOSF1	NM_202758,	chr18	670323	712517	-
	NM_001126123				
	NM_017512				
YES1	NM_005433	chr18	721591	812327	-
ADCYAP1	NM_001099733	chr18	904943	912173	+
	NM_001117				
METTL4	NM_022840	chr18	2537523	2571489	-
NDC80	NM_006101	chr18	2571509	2616634	+
SMCHD1	NM_015295	chr18	2655885	2805015	+
EMILIN2	NM_032048	chr18	2847027	2914090	+
LPIN2	NM_014646	chr18	2916991	3011945	-
MYOM1	NM_003803	chr18	3066804	3220106	-
	NM_019856				
MYL12A	NM_006471	chr18	3247527	3256234	+
MYL12B	NM_033546	chr18	3262110	3278282	+
	NM_001144944				
	NM_001144945				
TGIF1	NM_174886	chr18	3412071	3458406	+
	NM_173207				
	NM_173209				
	NM_173208				
	NM_003244,				
	NM_170695				
	NM_173210				
	NM_173211				
DLGAP1	NM_001003809	chr18	3498836	3845296	-
	NM_004746

**Table 2:** Genes and their genomic location within the deleted segment at 18q

Gene Symbol	Gene ID	Chromosome	Start Position	End Position	Strand
ZNF516	NM_014643	chr18	74069636	74207146	-
	NM_007345				
MBP	NM_001025081	chr18	74690788	74729055	-
	NM_001025090				
	NM_002385				
	NM_001025101				
	NM_001025100				
GALR1	NM_001480	chr18	74962007	74982096	+
ATP9B	NM_198531	chr18	76829396	77138282	+
NFATC1	NM_172390	chr18	77155771	77228177	+
	NM_006162				
	NM_172388				
	NM_172387				
	NM_172389				
CTDP1	NM_004715	chr18	77439800	77514510	+
	NM_048368				
	NM_001202504				
PQLC1	NM_001146343	chr18	77662419	77711653	-
	NM_001146345				
	NM_025078				
HSBP1L1	NM_001136180	chr18	77724581	77730822	+
TXNL4A	NM_006701	chr18	77732866	77748532	-
RBFA	NM_001171967	chr18	77794345	77810652	+
	NM_024805				
ADNP2	NM_014913	chr18	77866914	77898228	+
PARD6G	NM_032510	chr18	77915116	78005397	-

## Discussion

Ring chromosome 18 syndrome is a rare human cytogenetic abnormality. The syndrome is formed from breakage of both ends of the chromosome and the break ends generate a ring chromosome. The phenotype with r(18) syndrome is highly variable and depends on the combination of 18p- syndrome and 18q- syndrome [7]. The deletion of the short arm of chromosome 18 became a well-known chromosomal aberration after first discovery by de Grouchy in 1963 [2]. In 2009, Patricia et al. analyzed 18q in a high resolution level using aCGH, although they clarified the detailed breakpoint location, the deleted genes result from breakage of 18q were not able to be identified [8]. Normally, people use conventional karyotyping, FISH or aCGH to analysis chromosome aberrations, however, these methods have their limitations of revealing responsible critical genes and clarifying the genotype-phenotype correlations. Whole-genome low-coverage sequencing analysis could solve these problems at a base-level resolution.

Immunoglobulin A deficiency is frequently associated with ring chromosome 18 syndrome [9]. However, IgA deficiency was not noted in our patient, and further our patient appears features of central autoimmune hypothyroidism and small pituitary glands. The pituitary glands of our patient appeared morphologically small on head magnetic resonance imaging, while the thyroid showed morphologically normal on ultrasound. After receiving 10 months hormone therapy (levothyroxine), the IGF-1, T3 and T4 levels were still low, indicating that small pituitary invoked some functional defects, which resulted in the negative feedback failure of Hypothalamus-hypophysis-thyroid axis (HHTA).

There were totally 31 genes deleted at the del(18p) and del(18q) region. Some of them are very important for the physiological activity of the cells. Such as the USP14 gene that encodes a member of the ubiquitin-specific processing (UBP) family of proteases that is a deubiquitinating enzyme (DUB). Mice with a mutation that results in reduced expression of the ortholog of this protein are retarded for growth [10]. Gripp et al. [11] concluded that TGIF1 links the NODAL signaling pathway to the bifurcation of the human forebrain and the establishment of ventral midline structures. The GALR1 gene is widely expressed in the brain and spinal cord, as well as in peripheral sites such as the small intestine and heart [12]. Mutations in CTDP1 gene are associated with congenital cataracts, facial dysmorphism and neuropathy syndrome (CCFDN) [13]. So that, the inactivity of these genes may results to neurodevelopment, craniofacial appearance, oral manifestations and brain development anomalies.

In this report, We have presented a ring chromosome 18 patient with two heterozygous deletions of 3.88 Mb and 4.83 Mb indentified by whole-genome low-coverage sequencing method. The deletion of the genes and ring closure of chromosome 18 contribute to the clinical picture of dysmorphogenesis and mental retardation. Detailed breakpoints location and deleted genes identification help to estimate the risk of the disease in the future. At the same time, further studies are needed to delineate the function of responsible critical genes and clarify the genotype-phenotype correlations. The report here demonstrated the feasibility of next-generation sequencing technologies for chromosomal structural variation including ring chromosome in an efficient and cost effective way, which would improve the detection and prediction of genotype and phenotypic outcomes to direct postnatal medical care.

## Conclusions

In conclusion, we analyzed a ring chromosome 18 patient in China by whole-genome low-coverage sequencing method for the first time. We described the full profile of clinical examination, genetic characterizations, and clinical treatment report. We localized the genomic breakpoints as well as identified the deleted genes. Detailed breakpoints location and deleted genes identification help to estimate the risk of the disease in the future. The report here demonstrated the feasibility of next-generation sequencing technologies for chromosomal structural variation including ring chromosome in an efficient and cost effective way, which would improve the detection and prediction of genotype and phenotypic outcomes to direct postnatal medical care.

## Consent

Written informed consent was obtained in accordance with the Institutional Review Board of Wuhan Maternal and Child Health Hospital and the Declaration of Helsinki. The parents permitted the publication of the case, their clinical details and images.

## Additional file

Additional file 1: Table S1. The serological examination results. (DOCX 13 kb)
