# Supplementary material for: Breakpoints and deleted genes identification of ring chromosome 18 in a Chinese girl by whole-genome low-coverage sequencing: a case report study
Source: BMC Med Genet. 2016 Jul 22;17:49. doi: 10.1186/s12881-016-0307-1 (PMC4957311; doi:10.1186/s12881-016-0307-1)
Supplement: Additional file 1: Table S1. — The serological examination results. (DOCX 13 kb) [file 12881_2016_307_MOESM1_ESM.docx]

**Table S1. The serological examination results**

| **Biochemistry detection items** | **Result** | **Reference ranges** | **Unit of measurement** |
| --- | --- | --- | --- |
| **Liver and kidney function analysis** |  |  |  |
| Total cholesterol | 6.03 | 2.8-4.8 | mmol/l |
| Low density lipoprotein (LDL) | 4.27 |  | mmol/l |
| Lipoprotein-α | 520.2 | 0-300 | mg/l |
| High density lipoprotein(HDL) | 1.17 | 0.9-1.74 | mmol/l |
| Triglyceride | 0.79 |  | mmol/l |
| **Thyroid function analysis** |  |  |  |
| Thyroid stimulating hormone (TSH) | 3.409 | 0.51-6.27 | μIU/ml |
| Triiodothyronine (T3) | 1.07 | 1.26-3.50 | nmol/l |
| Tyroxine (T4) | 58.4 | 65.17-159.33 | nmol/l |
| Thyroid autoantibodies | positive |  |  |
| TPO-Ab | 1300 | 0-60 | U/ml |
| TG-Ab | 500 | 0-60 | U/ml |
| IGF-1 | 25 | 52-297 | ng/ml |
| IGF-BP3 | 0.54 | 1.3-5.6 | μg/ml |
| CORT (8AM) | 456.92 | 118.6-618.0 | nmol/l |
| CORT (8PM) | 179.95 | 85.3-459.6 | nmol/l |
| ACTH | 23.4 | 0-46 | pg/ml |
| IgA | 1.82 | 0.33-1.78 | g/L |
| CD3 | 72 | 38.56-70.0 | % |
| CD8+T | 43 | 13.24-38.53 | % |
